# Adipose Tissue, Regeneration, and Skin Health: The Next Regenerative Frontier

**DOI:** 10.1093/asjof/ojae117

**Published:** 2024-11-26

**Authors:** Alan D Widgerow

## Abstract

Adipose tissue, or fat compartments, has long been considered a storage depot and an energy source. However, a large part of new research, starting with the discovery of adipose-derived stem cells, has redirected this thinking toward the tremendous regenerative capacity that adipose tissue possesses when it is healthy. This has resulted in multiple technologies being explored with fat as a basis or with fat as a target aiming at the stimulation of new small hyperplastic adipose cells exuding adipokines and encouraging the proliferation of a whole host of progenitor cells that can have positive effects on many organ systems. One of these organ systems is skin, and there is a direct correlation with various fat compartments and skin health. Dermal fat tissue, also known as dermal white adipose tissue, is one such compartment that originates from dermal preadipocytes transdifferentiating into adipocytes and progenitor adipose cells under the right cues. The author of this paper discusses these potential cues, including injectable fillers, fat grafts, and topical formulations, and their capacity to impact skin health through the generation of healthy fat tissue. In addition, small molecules such as glucagon-like peptide-1 peptides and their impact on fat tissue are discussed. Adipose tissue is being recognized as the next regenerative frontier with exciting prospects ahead.

**Level of Evidence: 5 (Therapeutic):**

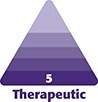

It can be said that, over the years, fat has received a “bad wrap.” At every opportunity, patients endeavored to remove this tissue through suction, surgery, or devices designed to indirectly injure and induce fat destruction. This is not surprising because western diets and sedentary lifestyles brought about elements of obesity, bodily changes, and metabolic consequences that were directly linked to excess adipose tissue in particular compartments.^1^ Evolutionary adaptation enabled mammals to store large amounts of energy in the form of fat. But this need for energy gradually decreased and the calorie requirements were oversatisfied, creating large fat depots that came with their own problems, particularly and inexplicably when these fat depots were located in visceral fatty deposits.^1^

“Bad fat” exudes inflammatory cytokines and reactive oxygen species (ROS), creates senescent cells, and negatively affects the autophagic processes.^2^ “Good fat,” on the other hand, can be the most regenerative of tissues, the new small healthy fat cells promoting multiple nonmetabolic regenerative effects in the skin often from compartments previously unrecognized, such as the dermal white adipose tissue (dWAT) situated at the base of the hair follicle and seeded throughout the dermis.^2-4^ Interestingly, the lineage differentiation and functionality of dWAT cells is different from that of other fat depots with newly defined roles in hair follicle regulation,^5^ wound healing,^6,7^ aging and fat fibrosis,^5,8^ immunity, antimicrobial peptide cathelicidin formation,^9,10^ and thermoregulation.^11^ A small, hard to discern, traditionally ignored fat compartment with immense functionality and regenerative potential has now become an important target in multiple physiologic processes (Figure 1).

**Figure 1.**
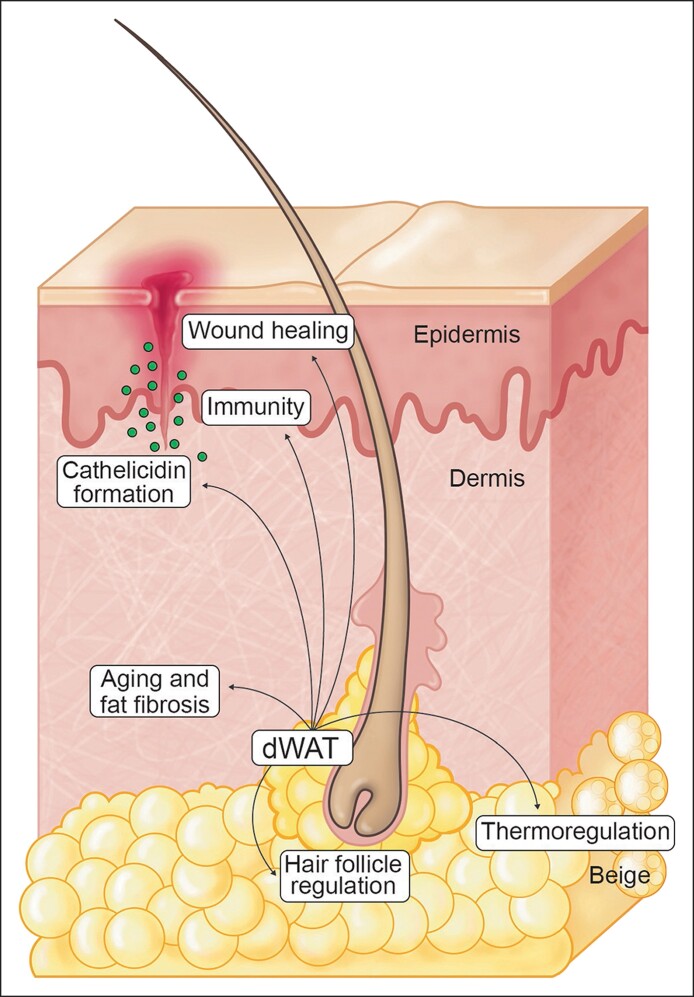
The dermal white adipose tissue is a small fatty compartment mostly recognizable as that tissue “cupping” the bottom part of the hair follicle. However, it is likely that these cells may arise from cells scattered throughout the dermis in the form of preadipocytes that can be indistinguishable from fibroblasts. The formed dermal white adipose tissue (dWAT) has multiple functional capacities—skin health from adipokine stimulation (aged skin, fibrosed dWAT); wound healing; cathelicidin formation against infection; hair follicle regulation; and thermoregulation.

In addition to these unique characteristics of dWAT, there is also a special relationship between these cells and dermal fibroblasts. They share common precursor cells in the dermis,^1^ and dermal fibroblasts are characterized by subpopulations that have unique properties associated with their location. The superficial papillary layer with a high density of fibroblasts produces soft connective tissue consisting of immature collagen (mainly Type 3) and elastin, whereas the deeper reticular layers contain a lower density of fibroblasts that produce thicker, more mature, and stable collagen fibers (mainly Type 1).^12,13^ Thus, papillary and reticular fibroblasts have distinct identities, with the deeper fibroblasts being almost indistinguishable from preadipocytes, demonstrating the interdependence of these 2 cell types—the preadipocyte can move toward a fibroblast or myofibroblast (fibrotic circumstance) or in the direction of the adipocyte (dWAT regenerative circumstance) depending on the signaling and milieu of the extracellular matrix (ECM)^13^ (Figure 2).

**Figure 2.**
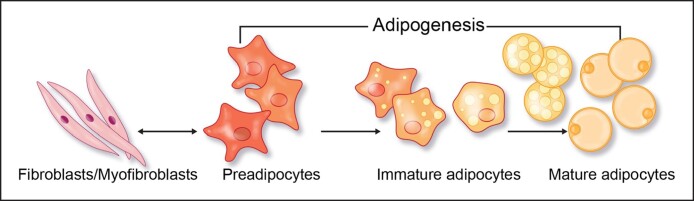
Preadipocytes are the precursors to adipocytes and circulate in the dermis waiting on cues for differentiation into adipocytes. They exhibit extreme plasticity, reverting to fibroblasts or myofibroblasts under stresses such as ultraviolet light or protracted inflammation (scarring), or they can transdifferentiate into adipocytes collecting lipid particles as they evolve. From a regenerative aspect and skin health, this transdifferentiation into adipocytes promotes adipokine production with positive effects on the skin.

In addition, it has been demonstrated that enlarged adipocytes significantly suppress the synthetic activity of fibroblasts.^14^ This dynamic reciprocity between fat tissue and fibroblasts has significant implications in regenerative processes.

The practical implications of these anatomic and biologic nuances are very relevant to skin health and may clarify many effects seen with various intervention procedures or topical applications. Thus, skin aging may directly link to fibrotic changes within the dWAT compartment, creating superficial atrophy, fine lines, and a lack of adipokines impacting skin health. Fillers may have varied effects depending on where they are injected and where the agent disperses—deep reticular dermis fibroblast stretching preferentially secreting Coll 1 as opposed to dispersion in the papillary dermis where fibroblasts would preferentially produce Coll 3. Topical products that specifically target fat tissue, particularly dWAT, could aid in skin plumping, adipokine elaboration, and autophagic removal of injured fat tissue. Fat grafting processes also have unique sequential phases leading to survival or death, and the potential regenerative properties of the graft itself have effects on skin health. Body contouring surgery has direct effects on fat tissue, with disruption causing an elaboration of lipid droplets from damaged fat cells, creating localized inflammation and skin induration. Lastly, the revolution taking place with weight loss agents, particularly glucagon-like peptide-1 (GLP-1)-related molecules, has a direct impact on fat tissue and ultimately skin health. Advancements in science have provided clarity to many previously unanswered questions related to therapies and aesthetic changes with aging and photodamage. Many of these, if not most, relate to the important physiological and regenerative capacities of fat tissue. The author of this paper covers the advancement of scientific understanding in these areas.

## METHODS

A comprehensive literature review was conducted in September 2024 to establish research carried out defining the possible relationship between fat tissue compartments, fat regeneration, and skin health. The National Library of Medicine database (PubMed) was used as the author’s primary source for the search, including papers up to September 2024. The search strategy was wide-ranging, which included “dermal white adipose tissue and skin health,” “adipose derived stem cells and skin health,” “fat regeneration and skin health,” “GLP-1RA and skin health,” “topical formulations and fat regeneration,” “skin aging and fat,” and “injectable fillers and fat.” The search was supplemented by research studies and previous publications by the author as well as relevant publications in related subjects, such as fat grafting, autophagy, adipokines, and skin health. Publications allied to fat regeneration but not directly relating to skin health were excluded.

### Skin Aging and Adipose Tissue

As opposed to other fat depots, the subcutaneous fat compartment (sWAT) and that seeded within the dermis, the dWAT, both thin with age.^2^ These compartments are rich in stem cells, adipokines, and immune cells, and the loss of these cells can have consequences on the dermal ECM and skin function. This decrease in adipocyte progenitors with defective differentiation into adipose-derived stem cells (ADSCs) results in increased transformation to senescent cells and limited or altered adipocytokine numbers and functionality.^2^ In addition, a commonly overlooked phenomenon is the age-related, but more importantly, photodamage-induced, fibrotic transformation of the small dWAT compartments and defective adipokine production that results in fat tissue atrophy, fine lines, and compromised skin health.^8,15^ This adipocyte myofibroblast transition has created a focus of interest in targeting dWAT as a means of impacting aging consequences and reversing ECM changes.^15^ It is likely that there are preadipocytes constantly residing within the dermis, unidentifiable or indistinguishable from fibroblasts that are on the lookout for signals that drive transition either to the fibrotic fibroblast/myofibroblast phenotype or toward a regenerative adipogenic pathway (Figure 2). The push toward the regenerative alternative using new approaches will result from a recognition of that science.

Thus, subcutaneous and dWAT fat are altered and diminished with aging, but a discussion on dWAT would be incomplete without noting the other significant functions that dWAT serves. The fibroblast-like preadipocytes residing in the dermis can undergo rapid proliferation and differentiation into adipocytes. As part of that process of adipogenesis, the cells develop the capacity to synthesize antimicrobial peptide cathelicidin, particularly in response to infection (acne, impetigo, etc).^2^ This immune function plays a direct role in maintaining healthy skin. In cold environments, as part of thermoregulatory control, dWAT can create adipocytes that are more “beige” in nature with increased mitochondria and energy production dissipating energy into heat.^2^ With its close proximity to the hair follicle, it is no surprise that dWAT appears to be influenced (and vice versa) by the hair follicle cycle, with prominent dWAT thickening occurring around anagen hair and subsequent thinning when hair follicles transition through catagen to telogen.^3^ This has created immense interest in utilizing this specialized fat compartment in hair follicle physiology (Figure 1).

Although all these ancillary functions of dWAT are fascinating and exciting to explore scientifically, from a skin aging perspective, the practical reality is that photodamage, and to a lesser extent, aging, cause the adipocyte to myofibroblast transition described previously. This results in fibrotic change to dWAT and sWAT with decreased adipokine production. How do adipokines relate to skin health?

Adipokines are small-molecular-weight, biologically active proteins produced by adipocytes (and some other cells). Although there are a host of proinflammatory and anti-inflammatory adipokines that may be involved in many skin diseases,^16^ when it comes to skin health, adiponectin has been recognized as a dominant protein involved in skin health and homeostasis.^17^

Adiponectin, an adipocyte-derived cytokine, is antidiabetic and has numerous properties. Related to skin health, it impacts multiple cell lines—keratinocytes, fibroblasts, melanocytes, and immune cells. It promotes the migration and proliferation of keratinocytes, impacts lipid synthesis and barrier integrity, and has ROS-quenching abilities related to photodamage in keratinocytes.^17^ It has been reported to boost hyaluronic acid (HA) and collagen production and decrease collagenase - matrix metalloproteinase-1 (MMP-1)- production in fibroblasts, while also having concomitant antifibrotic effects balancing collagen production.^17^ It also appears to inhibit melanin synthesis in melanocytes and encourage M1 to M2 macrophage polarization with an anti-inflammatory regenerative effect.^17^ Thus, from a skin health perspective, increased HA, collagen, decreased MMP-1, M2 promotion, normalized pigmentation, and improved barrier function are all beneficial effects for skin health and inhibition of skin aging. As such, adiponectin could be used as a molecular biomarker for skin health.

To a lesser extent, leptin is involved in aging skin health. Leptin is an adipokine synthesized and secreted by white adipose tissue and is involved as a main regulator of food intake, body mass, and metabolism.^18^ Ultraviolet light decreases the expression of adiponectin and leptin or its receptors, creating leptin dysfunction and increased levels of collagenase (MMP-1), resulting in collagen breakdown.^19^

When considering these positive effects related to adipokines and adiponectin in particular, combined with the regenerative potential of preadipocytes and ADSCs and a host of progenitor cells found within the fat tissue compartment, it is not surprising that manufacturers of topicals and fillers are examining the potential effects of these agents on fat tissue to compound their antiaging effects. Of course, proponents of fat grafting on its own or in combination with rejuvenation procedures have long reported concomitant skin health benefits with grafting procedures. The reasons for this are now becoming more apparent.

### Injectable Fillers and the Relationship to Adipose Tissue

Restoring the fat compartment has become a recognized strategy in combating aging skin. Fat grafting is a logical solution to this problem and is commonly and successfully performed, but results can be unpredictable, and the process does involve a surgical procedure.^20^ Thus, soft tissue filler injections have become a popular alternative, although not always with fat tissue stimulation as a primary objective. This trend is changing, with more attention being given to potential adipogenic effects from soft-tissue fillers. Aside from actual fat grafting, the most focused fat tissue replacement fillers have been those with inherent fat tissue components. These may be derived from cadaver tissue, such as allograft adipose matrix (AAM, Renuva; MTF Biologics, Edison, NJ), an off-the-shelf, soft tissue matrix used for cushioning and volume restoration.^21^ Thisfiller material constitutes a fat matrix replacement rather than a traditional filler, but the graft retention rate is still a problem with ∼30% survival of that injected in studies in the malar region.^21^ That noted, autologous fat grafting retention rates are highly varied and do not differ very much from these recorded figures.^20^

Thus, retention rates are limited with current autologous fat grafting and fat matrix component replacement, but what effect can soft tissue fillers have on residual host fat tissue and how can that affect skin health? The first indication that fat components could affect skin health related to the “nanofat” concept. Introduced by Tonnard et al in 2013, mechanical emulsification of fat, while destroying many fat cells, appeared to retain progenitor cells, which may have contributed to the positive effect on skin health.^22^ Since that time, many variations in nanofat preparation have been made, regenerative progenitor cells have been identified, and this nonvolumization effect of the components of fat tissue has been identified as important for skin health.^23,24^ The question then remains: Should we not be attempting to impact host fat tissue with soft tissue fillers to tap into this regenerative effect, releasing adipokines and altering skin homeostasis?

To this end, relatively inert HA-based soft tissue fillers were hypothesized to stimulate ADSCs and increase adipose cell proliferation either from their effect on surrounding ADSCs or by direct effects of HA on the adipocytes.^15,25-27^ As evidence for direct effects of HA on adipocytes, the authors of 1 study detailed increased differentiation of preadipocytes to adipocytes, decreased lipolysis, and increased adiponectin secretion during a long-term culture protocol when exposed to cross-linked HA.^26^ Although it has been recognized that mechanical stress converted to biochemical signaling, mechanotransduction, has a direct effect on stretched fibroblast increasing collagen production, there may be added effects on ADSCs from different physical factors.^25^ HA fillers appear to cause ADSC stimulation, which may be able to stimulate fat tissue expansion and induce long-term effects.^25^ Another important speculative consideration is that this proliferative and differentiation response of ADSCs is dependent on the metabolic health of the individual, with diabetic patients often manifesting a dysfunction of ADSCs and an inability to respond to stimuli as normal patients would.^25,28^ With the above noted, this HA influence on adipose tissue is relatively unproven and indirect in its approach. More importantly, new-generation soft tissue fillers that have active components that directly affect fat tissue, stimulating healthy new fat cells and increasing adipokine release, would herald new, truly regenerative soft tissue fillers. Work is ongoing in this sphere.

The above discussion and previous publications suggest that the biostimulatory soft tissue filler paradigm should be shifted from neocollagenesis to adipogenesis in the dermis and subcutaneous tissue, taking full advantage of the powerful regenerative capacity of adipose tissue.

### Fat Grafting and Skin Health

Over the past 2 decades, fat grafting has become an integral part of facial rejuvenation, with almost every facelift procedure including a fat additive component. Much of this logic stems from early work done by Tonnard et al, displaying the inherent capacity of fat tissue, even in its emulsified nanofat state, to have significant effects on skin health.^22,29^ In the case of nanofat, it has been demonstrated that the mechanical sheer stress, although destroying a large number of fat cells, also creates a potent progenitor cellular phenotype that can have significant regenerative effects.^23^ In addition to nanofat variation, fat grafting in its cleaned macro form, aside from potential volumization, also appears to offer various regenerative components, from stromal vascular fraction (SVF) containing pericytes, endothelial progenitor cells, ADSCs, and multiple other regenerative cells to the secretome of adipokines elaborated by these cells, including adiponectin and leptin described above.^30,31^

Thus, there is general consensus that fat grafting with all its variations, is an integral part of facial rejuvenation processes today. However, there is still inconsistency in fat graft survival particularly when the primary objective is volumization. To this end, an analysis of fat graft survival reveals a multitude of steps by which fat grafts can be manipulated or augmented in an effort to improve survival. As opposed to early thinking that fat grafts simply augmented or replaced the recipient area grafted, it soon became apparent that a large part of the graft does, in fact, not survive, and that replacement with new tissue is generated by progenitor cell effects on the host tissue. The sequences of tissue replacement that takes place with fat grafting procedures can be manipulated using various strategies. In 2015, we coined the phrase “strategic sequences” in fat grafting to describe the potential interventions that could be undertaken to improve fat graft survival.^31^ These included enrichment with stromal vascular fraction or ADSCs, stimulation of autophagic processes (topically or systemically), introduction of a scaffold ECM to the mix, and so on.^31^ Cohen et al have added nuances to the fat grafting procedure, taking advantage of the regenerative properties of fat but using facial topography aging changes to select varying fat graft sizes (milli, micro, and nano) to replace losses of fat occurring at different depths and in different anatomic regions.^32,33^

It is apparent that fat grafting in one form or another is here to stay. Adipose tissue is no longer considered just a lipid storage depot, but rather an endocrine organ with major regenerative potential related to its adipokine profile and a multitude of progenitor cells. The advancements in grafting are likely to manifest from additional sophisticated devices used in the preparation of the grafts, enrichment of traditional grafts with added cellular components (SVF and ADSCs), and possible incorporation with novel scaffolds to ensure large volume replacements (breast augmentation as an example). Skin health and fat grafting may focus on the generation of adipokines such as adiponectin rather than volumization, and it is likely that the component within the graft that maximally generates the secretome will be the ideal fat graft for combating aged skin.

### Topical Formulations, Adipose Tissue, and Skin Health

Taking into consideration these scientific nuances related to adipose tissue, it is evident that this organ system can significantly impact skin health. It is therefore surprising that more companies in the topical formulation space have not dedicated time, effort, and research geared toward influencing adipose tissue function through topical formulations. In my capacity (disclaimer) as Chief Scientific Officer of Galderma (Galderma Laboratories, Dallas, TX), and more particularly related to formulations in the ALASTIN Skincare range (ALASTIN Skincare, Carlsbad, CA), I have directed multiple studies related to topical formulations and adipose tissue metabolism. In general terms, I will discuss some of the interesting scientific aspects and new avenues that we have explored in this area and the interesting new information we have gleaned from these studies.

**Figure 3.**
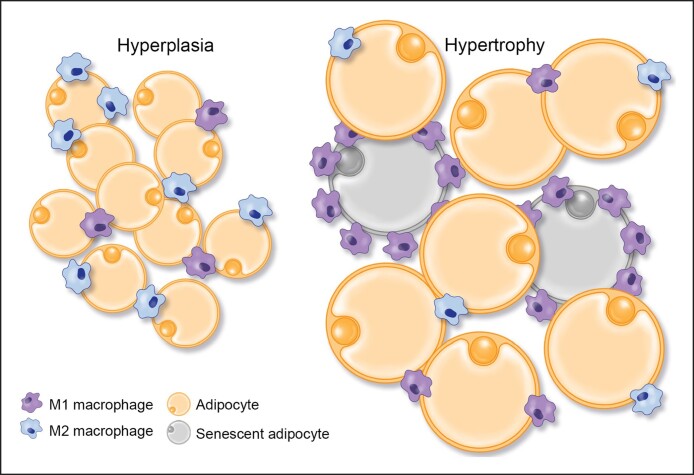
Hypertrophic large fat cells (such as those seen in diabetic patients) tend to elaborate proinflammatory cytokines and are associated with M1 inflammatory macrophages, increased senescent cells, and decreased adiponectin secretion. This is in direct contrast to young small fat cells that elaborate good levels of adiponectin and other healthy adipokines, associate with M2 regenerative macrophages, and appear to represent the regenerative component of fat tissue.

Before we discuss the background of product formulations, additional relevant adipose science, is relevant. As previously noted, large hypertrophic adipocytes associated with obesity elaborate inflammatory cytokines and constitute an unhealthy background metabolic milieu representative of diabetes and other disease states. This is well recognized. What is less recognized is the effect that these hypertrophic adipocytes may have on surrounding fibroblasts. When in culture, these adipocytes secrete low levels of adiponectin, downregulate collagen and elastin genes, and upregulate MMP13 and MMP9 in surrounding fibroblasts, decreasing collagen and elastin formation and creating a dysfunctional ECM.^14,34^ This is not the case with small adipocytes. It appears that this alteration in fibroblast function may be related to the release of free fatty acids (FFAs), particularly palmitic acid, by the hypertrophic adipocytes.^14^ The hypertrophic adipocytes elaborate inflammatory cytokines and are associated with a majority of M1 inflammatory-type macrophages, whereas small healthy adipocytes do not have that effect and are associated predominantly with M2 regenerative-type macrophages (Figure 3).

Thus, the major goal of a topical formulation targeting adipose tissue has as its main aim the creation of small healthy adipocytes.

Restorative Skin Complex (ALASTIN Skincare) was formulated as a skin maintenance program aimed at combating aged photodamaged skin. Of relevance, the formulation includes constituents aimed at volumizing and plumping the skin by stimulating new healthy fat cells. In this formulation, a PGC1a stimulator (peroxisome proliferator-activated receptor-gamma—PPARg—coactivator 1 alpha) is included. PGC1a is a transcriptional coactivator that strongly induces the differentiation of preadipocytes of mesenchymal origin into white adipocytes under the influence of PPARg.^34,35^ The young newly formed adipocytes formed under these conditions would be expected to generate good elastin formation and increased adiponectin levels with subsequent improvement in corneocyte barrier appearance, both demonstrated in biopsy analyses.^35^

Although a large part of the discussion has focused on fat buildup or adipogenesis, and ways to stimulate the process, the opposite, fat breakdown or adipocytolysis, also has relevance to skin health, particularly related to surgical procedures. This first became apparent with noninvasive cryolipolysis, in which the crystallization of fat cells and loss of integrity resulted in the release of lipid droplets and FFAs. What was not immediately apparent was that these lipid droplets were extremely proinflammatory, releasing multitudes of cytokines and manifesting in the skin as local edema and induration that resolved once these fat droplets were absorbed.^36^ The challenge was to obtain the lipid droplets to be absorbed by macrophages, which are infinitely smaller than lipid droplets. The authors of a research generated with in vitro, gene expression and ex vivo studies uncovered a mechanism for breaking down these lipid droplets through a process of autophagy, or lipophagy in the case of fat. Autophagy is a sophisticated mechanism that enables recycling of large organelles and molecules by breaking them down in an enzymatic lysosomal process that enables their absorption, while generating energy in the process. When cells or organelles are damaged beyond repair, autophagy aids in the digestion process, and when they are not severely damaged, the process will use generated energy to replenish these cells and encourage survival^33,37^ (hence the importance in fat grafting as previously described). Using this newly described science as background, peptides with autophagic capacity were investigated and utilized in formulations directed at noninvasive fat reduction,^38^ and later, invasive body contouring surgery,^39,40^ with the purpose of efficiently removing lipid droplets with a resultant decrease in induration, edema, and fibrous banding of the skin demonstrated in multiple clinical trials.^40-44^

The work done related to topical treatments demonstrating its effect in some cases on subcutaneous fat created a new paradigm for topical treatment. By utilizing liposomes, allowing entry through hair follicle channels, direct access to dWAT and then access to underlying sWAT ensured almost instant access to the deep compartments from surface treatments of the skin.^38^ Thus, fat tissue has become a target for skin health, and topical formulations can play an important part in this regenerative process.

### GLP-1 Receptor Agonists, Fat Tissue, and Skin Health

GLP-1 receptor agonists (GLP-1RAs) represent a revolution in weight control and are here to stay. These peptides cause weight loss because of an apparent reduction in energy intake from decreased appetite, thought to be linked to direct and indirect effects on the brain.^45^ GLP-1 is a gastrointestinal hormone, usually produced after meals, stimulating insulin secretion and inhibiting glucagon release.^46^ After significant weight loss was observed in many patients treated for diabetes, the primary focus shifted to treatment for obesity in nondiabetic individuals.^46^ In many cases, this weight loss has been accompanied by changes in facial appearance, mainly involving depleted volume, but also by increased aging, sagging, and wrinkling—this has become so apparent in some cases that a descriptive term called “the Ozempic face” has been adopted.^47^

There is still much to learn related to these facial changes because it does not simply follow the logic of massive weight loss causing sagging skin—there appears to be chemical and biologic nuances to the peptides and their effects on adipose tissue components.^48^ The previous discussion on dWAT is also very relevant here, and it would appear that this specialized tissue with its unique progenitor cell collection may well be the focus of GLP-1 effects on the skin. dWAT, among other cells, is associated with a strong contingent of preadipocytes—these cells resembling fibroblasts are programmed to either revert to fibroblast-like cells or to transdifferentiate into adipocyte-like cells with all their various forms (ADSCs, pericytes, endothelial progenitor cells, etc.).^8,15^ The GLP-1 peptide appears to inhibit adipocyte differentiation and this effect may be limited to humans.^48,49^ It appears very likely that a similar mechanism to photodamage and aging may be taking place in dWAT under the influence of GLP-1RAs—that is, a regression of preadipocytes into fibroblasts and myofibroblasts—with fibrotic changes and thinning of the ECM with resultant fat atrophy, sagging, and wrinkling. However, this has yet to be proven, but now it is an opportune time to start looking for answers. Of course, the speed with which weight loss occurs, and the extent of the weight loss cannot be ignored, with faster and more drastic weight loss likely to create more dramatic effects. However, this is not always the case, and therefore, further inquiry is warranted.

An added factor that has been suggested as worth examining is the loss of estrogen related to the reduced dWAT and ADSCs. Estrogen is protective of skin health, and loss of adipose tissue (and ADSCs) is accompanied by decreased estrogen levels, which can be associated with significant skin aging.^47^ Lean muscle mass loss has also been reported with GLP-1RA use, but results have been varied in this regard.^47^

In an effort to combat and reverse the changes described, multiple solutions are being sought. Replacing with fat grafting, particularly stromal vascular fraction–enriched fat grafting,^30^ would seem like a logical approach to replace fat loss and re-encourage preadipocyte to adipocyte differentiation. However, this solution does involve a procedure and is rather more complex than the use of a soft tissue filler. In this regard, it is likely that soft tissue fillers that have regenerative active components (affecting ECM and fat components) rather than traditional biostimulatory fillers (collagen bias) will be important new alternatives. In new research on fillers such as Poly-L-Lactic Acid (PLLA), authors will focus on this indication. Combining fat-targeted fillers (boosters), together with topical fat–stimulating formulations (maintenance), is likely to provide a synergistic solution.

## CONCLUSIONS

It is apparent that fat is a highly regenerative organ and not simply a source for energy supply. The intimate relationship between fat and skin provides us with a new target to influence skin health. To this aim, many new treatment modalities are now looking to influence adipose tissue as a means of initiating regenerative changes in different layers of the skin and beyond. From the discussions above, it is likely that the next generation of aesthetic treatments will include the following:

- fat grafting with improved preparation and progenitor cellular–enriched components;
- soft tissue fillers with active components having direct effects on fat tissue including stimulation and adipokine elaboration;
- topical preparations designed specifically to target the stimulation of fat tissue components or removal of breakdown products where needed; and
- a combination of the above to combat fat loss in aging and following the use of systemic drugs, the best current example being GLP-1 products.

Adipose tissue will be at the center of the regenerative conversation in the next phase of aesthetic advancements.
